# Good Ambulance Work in Glasgow

**Published:** 1914-09-26

**Authors:** 


### Good Ambulance Work in Glasgow.

The St. Andrew's Ambulance Association, which has its headquarters at 176 West Regent Street, Glasgow, of which Dr. Donald Mackintosh, M.V.O., is chairman, has for the past thirty years conducted the entire ambulance transport service in Glasgow and the chief cities of Scotland. In Glasgow, the Association owns six motor waggons, one motor-ambulance limousine, and two horse-ambulance waggons. In the present national crisis the military authorities arranged with the Association for the service of its waggons and trained attendants to convey the wounded soldiers to hospital; already upwards of 400 have thus been transported. The Association's waggons are fitted with the most modern appliances for the conveyance of patients in comfort and safety, and they are at the call of the military and civil authorities both day and night.

### From the Station to Stobhill Military Hospital.

In connection with the arrival of wounded in Glasgow direct from the Front, it is gratifying to note the excellent work displayed by the Association in the transportation of the wounded from the hospital train at the railway platform in the grounds of Stobhill Military Hospital to the hospital itself on Sunday, September 20, 1914. The Association was informed by the military authorities that they expected two hospital trains to arrive from Southampton with 200 patients on Sunday evening between 9 p.m. and 10 p.m., and that their services would be required to transport the wounded. This request was willingly acceded to, and the staff were detailed for duty. At 9 p.m. the first hospital train arrived with 100 patients, and when the military authorities had taken the wounded from the carriages they were immediately handed over to the Association's officers, who placed them in the waggons and conveyed them to the hospital. The work was performed by three fully equipped motor waggons carrying an additional supply of stretchers, with three drivers and six ambulance officers. On arrival at the hospital orderlies took charge of the patients, the stretchers being left with them, while the waggons returned by a circular route to the station platform, and in this way 100 patients were comfortably in bed within an hour's time. At 11.25 a second train drew up at the platform, and the same procedure was carried out, only in this train the cases requiring to be carried on stretchers were fewer in number, and the work was performed in a shorter time owing to the valuable assistance rendered by private motor-cars arranged for by the secretary of the Scottish Automobile Club. The patients from this second train were all in the wards in thirty-five minutes.

It certainly reflects great credit on all concerned that in less than two hours' time, and by means of artificial light only, two hundred wounded soldiers were safely conveyed from the trains to the wards of the military hospital.

